# Chicago Medical Society

**Published:** 1883-08

**Authors:** 


ARTICLE VII.

CHICAGO MEDICAL SOCIETY.

The Chicago Medical Society held its semi-monthly meeting on the evening of June 4th, with Dr. D. W. Graham, the president, in the chair.

The following gentlemen were elected to full membership: Dr. N. S. Davis, Jr., Dr. J. M. Pilsbury, Dr. D. F. MacPherson, Dr. N. E. Rice.

After which Dr. R. Tilly exhibited under the microscope numerous bacteria removed from a (decayed) tooth while the patient was present before the members. The patient was deaf and the bacteria were present very extensively in his mouth. The doctor stated that the fungi may pierce the body of the teeth, but in the case before us this evening, he was unable to decide positively whether their presence was the cause or effect of the dental decomposition, but inclined to the former opinion. He had recently discovered them while treating the patient for the deafness with which he is afflicted.

In the discussion which followed Dr. W. W. Allport participated. He said the old theory for decayed teeth was acids acting on the lime of the teeth, but later theories now advanced as causes for dental decay were the "chemical," the "vital chemical," the "parasitical" and "electrical" theories. Instances were cited under his observation where the eye-sight and hearing were affected by decayed teeth, and the filling in a tooth, and where the removal of one or the other exciting cause remedied the eye or ear trouble.

Dr. Alonzo Bryan then presented an interesting paper on the subject of "Stammering," which is recognized as a functional disease. Several pathological points of paramount interest were brought to notice, in which the literature is meager, and the following extracts upon this topic were taken:

"This pathological condition may occur in persons of perfect physical integrity. It is the result of mal-habit, or mal-education of the nerve centers, presiding either immediately or remotely over the organs of articulation. The act of stammering usually consists in the attempt of a person to articulate an elementary sound while the vocal organs remain in the position proper for the utterance of the sound which precedes it in the same syllable. A traumatic lesion of the mouth may temporarily disable a person in certain forms of utterance. If so, these cases are confined usually to a single letter, and often to only a single word. The most stammerers are capable of stammering upon every letter of the alphabet and upon every word in the language. At times momentary vertigo may ensue, and tetanic action of a number of the facial muscles may occur simultaneously in the extreme stammerer. Stammering occurs vastly more often in children than in adults, more frequently, too, in the male, and more persistently in those of small intellectual caliber, and disappears more readily in persons of high or good intellectual capacity. Fear will enhance the difficulties of the stammerer, and so will decrease of the rapidity of nervous conduction, as cold. During the hot weather of summer, stammerers are often greatly improved. Buoyancy of spirits affords relief. Consciousness of the presence of superior and arrogant persons plunges the common stammerer into difficulty. The presence of a benign superior aids him. Vigorous mental excitement usually aids and relieves the stammerer. A sense of heartfelt joy often does away, temporarily, with all difficulty of utterance. Illustrative examples of lingual impediments were cited, of lecturers, of young ladies, and of himself, who used to be an inveterate stammerer, which were dwelt upon quite minutely. Among the causes of stammering mentioned were antecedent disease and pertussis; children recovering from extreme adynamia in acute diseases are liable to stammer afterwards. A large proportion of stammerers are made by imitating others, who mimic a sufferer from the malady. A large number take on their trouble by unconscious imitation. A child may imitate its brother, sister or parent. Cases that supervene in this manner are not so inveterate as those that arise through ridicule or through a malicious purpose. Children born of stammering parents usually stammer with great facility. In this it may be of transient duration. Stammering is not analagous to *aphasia* due to cerebral lesion.

The treatment carried out intelligently should usually be successful. The tendency is to spontaneous recovery. Total non-interference should be adopted in treating young children who have acquired the impediment by unconscious imitation. The child should be placed in circumstances of absolute freedom of general growth, physically and mentally. A warm climate enhances recovery by increasing the velocity of nervous condition. The self-confidence of a child should be cultivated, and it should be removed from all opportunity to imitate another stammerer. It should be taught music, dancing and calisthenic exercises.

The intelligent adult stammerer should be drilled in elementary articulation. His general culture should be carried to an extreme extent. A high form of will should be required on the part of a patient.

After its conclusion Dr. E. G. A. Trimble asked the author of the paper if stammering was apt to be accompanied by neurasthenia, and if, in hereditary cases, the hesitation generally occurred on the vowels or consonants. Answered: No relation to neurasthenia; and, secondly, that the hesitation most often occurred on the vowel sounds.

Dr. J. H. Etheridge thought well of the pathology as stated in the paper, and asked if singing a word would ease them, or to stop short and count 5, 15, or 20? Answered negatively.

A vote of thanks was tendered to Dr. Bryan, after which the society adjourned.

A goodly number of visitors were present, who stopped while en route to the American Medical Association, and afterward accompanied the party of delegates from this society to Cleveland.

L. H. M.

---

THE ninth annual meeting of the Tri-State Medical Association, will be held in Indianapolis, September 18th, 19th, and 20th. The work is already far advanced and the title of each paper should be sent in at once. Papers must not exceed 25 minutes.

It is also the rule that each physician who registers must be a member of a local or State society in good repute. All such are invited.

Notice of papers or cases to be presented may be sent to the chairman of the committee on programme, Dr. J. L. Thompson, Indianapolis; to the Secretary, Dr. G. W. Burton, Mitchell, Ind., or to the President Dr. Wm. Porter, St. Louis.

